# Immune dynamics throughout life in relation to sex hormones and perspectives gained from gender-affirming hormone therapy

**DOI:** 10.3389/fimmu.2024.1501364

**Published:** 2025-01-16

**Authors:** Ahmet Yalcinkaya, Rumeysa Yalcinkaya, Fabian Sardh, Nils Landegren

**Affiliations:** ^1^ Science for Life Laboratory, Department of Medical Biochemistry and Microbiology, Uppsala University, Uppsala, Sweden; ^2^ Department of Medical Biochemistry, Hacettepe University Faculty of Medicine, Ankara, Türkiye; ^3^ Department of Pediatric Infectious Diseases, Ankara Etlik City Hospital, Ankara, Türkiye; ^4^ Center for Molecular Medicine, Department of Medicine (Solna), Karolinska Institutet, Stockholm, Sweden

**Keywords:** immune system, sex difference, gender-affirming hormone therapy, androgens, estrogens, autoimmune diseases, infectious diseases

## Abstract

Biological sex is closely associated with the properties and extent of the immune response, with males and females showing different susceptibilities to diseases and variations in immunity. Androgens, predominantly in males, generally suppress immune responses, while estrogens, more abundant in females, tend to enhance immunity. It is also established that sex hormones at least partially explain sex biases in different diseases, particularly autoimmune diseases in females. These differences are influenced by hormonal, genetic, and environmental factors, and vary throughout life stages. The advent of gender-affirming hormone therapy offers a novel opportunity to study the immunological effects of sex hormones. Despite the limited studies on this topic, available research has revealed that testosterone therapy in transgender men may suppress certain immune functions, such as type I interferon responses, while increasing inflammation markers like TNF-α. Transgender women on estrogen therapy also experience alterations in coagulation-related and inflammatory characteristics. Furthermore, other possible alterations in immune regulation can be inferred from the assessment of inflammatory and autoimmune markers in transgender individuals receiving hormone therapy. Understanding the complex interactions between sex hormones and the immune system, particularly through the unique perspective offered by gender-affirming hormone therapies, may facilitate the development of targeted therapies for infections and autoimmune diseases while also improving healthcare outcomes for transgender individuals. Here we review immune dynamics throughout life in both sexes and provide a summary of novel findings drawn from studies exploring gender-affirming hormone therapy.

## 1 Introduction

The immune system combats internal and external threats by employing a multitude of tools that operate in strict unison to create a formidable, near-infallible machine, which not only detects and neutralizes the ‘non-self’ but also continuously (re)assesses its foes and (re)calibrates its approaches while unleashing its *force majeure* (1, 2). Despite its limitations that come in various forms (eg, genetic and acquired deficiencies or evasive infections) and the well-understood fact that it is not devoid of errors in target selection (ie, autoimmunity) (3, 4), the human immune system is a dynamic biological network that is more often than not triumphant in the evolutionary arms race against ever-changing internal and external threats.

Such a comprehensive organization with a battlefield that encompasses the whole organism requires systems-wide check-and-balance mechanisms (5). These control mechanisms are enforced and maintained by numerous regulators, including the multitude of factors that are directly or indirectly associated with biological sex. Males and females differ not only in their susceptibility to certain diseases but also in how their immune systems respond to infections, vaccines, cancers, and other conditions (6). These differences are influenced by a combination of genetic, hormonal, and environmental factors, with sex hormones being critical since they underlie the divergent characteristics of sex (7). Androgenic hormones are generally accepted to weaken immune response, while estrogenic hormones usually have the opposite effect (8–10). Nonetheless, it has been notoriously challenging to determine the degree to which these hormones alter immune functions, largely because it is almost impossible to account for all biases and confounders that emerge from comparing the two sexes. Age and developmental stage are other sub-characteristics that further complicate analyses since these properties alter the production and response to sex hormones.

The emergence of gender-affirming hormone therapy (GAHT) and its utilization in different age groups represents a unique opportunity to not only understand how transgender individuals respond to this treatment but to also examine the impact of sex hormones on immunity. Comprehension of the impacts of the primary sex hormones on immune functioning can yield essential knowledge that can facilitate the development of target-specific medical treatments in infections or autoimmune disease and improve healthcare strategies concerning transgender individuals.

## 2 Innate and adaptive defense

The immune system consists of both innate and adaptive defense mechanisms. Innate immunity acts as the first line of defense by responding to structures that are consistently unique to invading microorganisms, which are recognized by the pattern recognition receptors of primarily dendritic cells and macrophages (1). The macrophages are masters of phagocytosis and cell lysis, yielding a powerful initial response to outside insults. The innate response also induces cytokine release and is the principal source of inflammation (11). Despite their strong presence and early response, macrophages and the innate response as a whole cannot recognize or deal with all threats, necessitating deployment of the otherwise-specialized elements of the immune system. Cytokine release is one of the factors that activates these specialized systems, namely the adaptive immune response, which recognizes and eliminates pathogens and infected cells through specific cytokine secretion and antibody production, while also creating an archive of previously-recognized threats (12). Lymphocytes, with their multitude of sub-specialized cells, are the cornerstone of the adaptive immune response. They produce antibodies and employ cell-mediated immune responses, which are respectively the responsibilities of B and T lymphocytes (12).

## 3 Sex hormones

**Figure 1:**
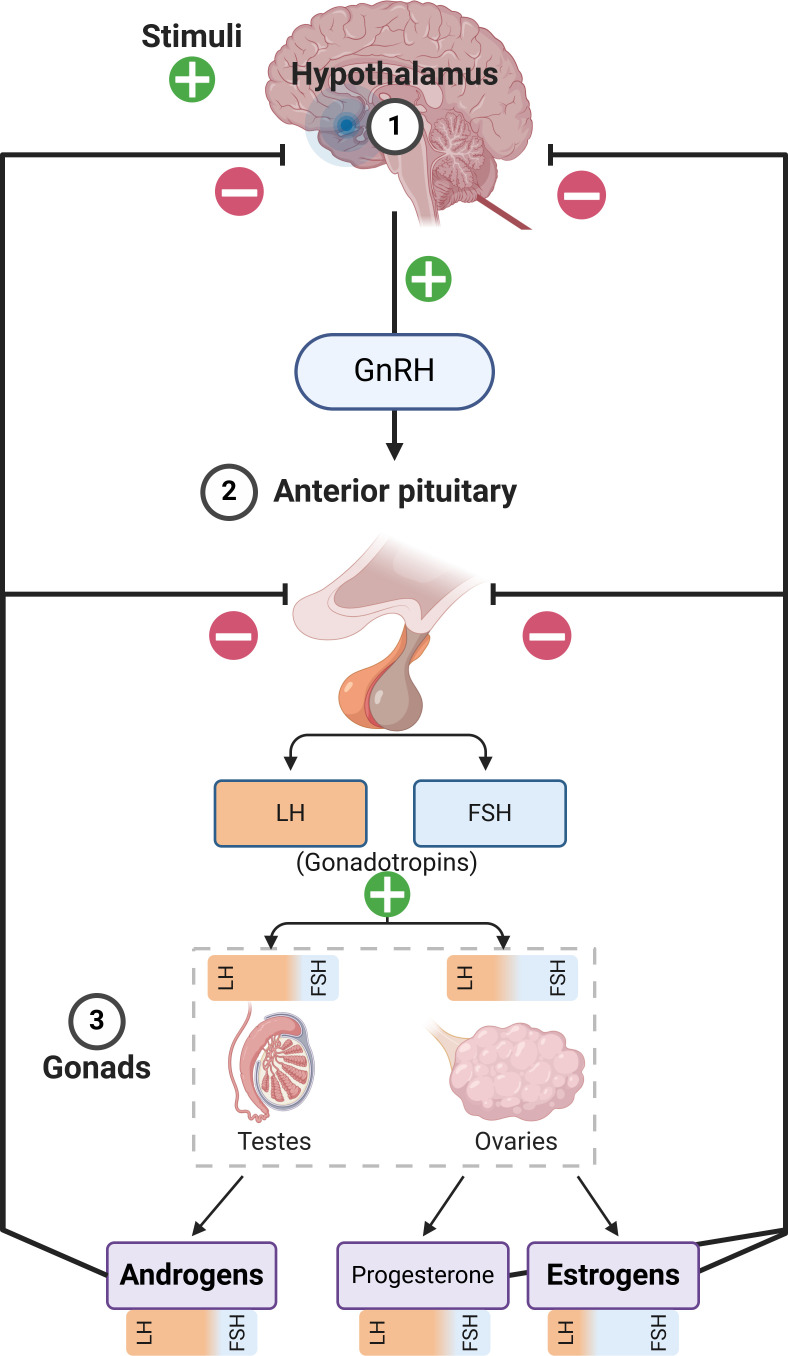
Summary of the Hypothalamic–pituitary–gonadal axis. It must be noted that androgens and estrogens are produced in both males and females in different tissues, at vastly different concentrations. The figure only describes the primary production pathways in order to emphasize sex-related differences with respect to hormonal production, while also showing the influence of luteinizing hormone (LH) and follicle stimulating hormone (FSH) on the gonads and the production of the respective hormones. GnRH, Gonadotropin-Releasing Hormone.

The classical categorization of sex hormones relies upon their different distributions in the sexes, with androgens dominating in males and estrogens dominating in females (**Figure 1**). Other categories with numerous members also exist. For instance, the progestogens include progesterone, which is a crucial hormone for females and contributes to numerous sex-related and unrelated functions (13). Irrespective of their sex-based distribution or physiological impact, all sex hormones are produced in healthy individuals and are structurally defined as steroids, which contain the 4-fused-ringed, 17-carbon steroid skeleton (“gonane”). This skeleton provides the fundamental basis for the unique physical and physiological properties of sex hormones, facilitating their transport, permeability, recognition, and functions (14, 15). Sex hormones exert their biological effects by binding to nuclear receptors, which function as ligand-activated transcription factors. Upon hormone binding, these receptors undergo conformational changes, dimerization, translocate to the nucleus, and bind to specific hormone response elements within the genome, thereby regulating the expression of target genes (16).

## 4 Sex-related immune differences

Sex-specific differences in susceptibility to autoimmune diseases, certain cancers, and infectious diseases have been documented widely. It is accepted that the evolutionary basis for this difference is the different requirements of the male and female immune systems –due to specific challenges unique to each sex. In particular, the female immune system needs to survive the immunosuppressive pregnancy period to create offspring while ensuring that the metabolic cost of maintaining the immune system is not too high, and also, immune response must be flexible enough to allow for pregnancy (17, 18). At baseline, females have stronger innate and adaptive immune responses compared to males, leading to faster infection clearance than males, better vaccination outcomes, and more potent serological response (19). This advantage is not solely confined to the urgency of the response either; males suffer from higher mortality rates after infection, even when adjusted for age, whereas females often mount stronger humoral immune responses, cytokine production, and T cell response after immune challenge (20), which likely explains their higher survival rates following infectious diseases (21).

### 4.1 Circulatory cells

A well-established method for assessing immunological states or properties is the characterization of circulating immune cells. Sex influences the composition of circulating white blood cells (leukocytes), either through sex hormone-related or independent mechanisms. Aggregated data from studies evaluating responses to different pathogens show that the number and activities of cells generating the innate immune response, including macrophages and dendritic cells, are higher in females relative to males (22, 23). Of note, the immune response is predominantly driven by type 1 helper T cells (Th1) and cellular immunity in males, while the female sex exhibits superior properties regarding B and T cell maturation and a stronger antibody response that is largely attributable to the dominance of type 2 helper T cells (Th2) (23, 24). Another subset of T helpers that has strong evidence of sex hormone-based modulation is the Th17 subset, which is downregulated by both estrogens and androgens (25) and is involved in the pathophysiology of autoimmune diseases through various mechanisms including their production of pro-inflammatory cytokines that sustain/prolong inflammation (26, 27). Flow cytometric immunophenotyping of healthy volunteers reveal higher levels of CD4+ T cells in females, which may indicate enhanced thymic activity –an organ with profound effects on lymphocytes and autoimmunity (28). The elimination of autoreactive T cells in the thymus via negative T-cell selection may be less stringent in females compared to males. The autoimmune regulator (*AIRE*) gene plays a critical role in this process by promoting the expression of tissue-specific antigens in medullary thymic epithelial cells, which are then presented to naïve T cells (29). Sex hormones drive *AIRE* expression in medullary thymic epithelial cells, and studies of human thymic samples and mouse models have revealed that AIRE expression is higher in males compared to females, partially explaining the divergence in developing autoimmune disease (30, 31). The sex differences also extend to CD19+ B-cells, regulatory T cells, plasma cells, and both naïve CD8+ and mucosa-associated invariant T-cells (32). On the other hand, females generally have lower absolute or relative levels of monocytes, myeloid cells, and lower absolute counts of natural killer (NK) cells than males (33). Furthermore, the adaptive immune response shows a more apparent sexual difference in antibody responses, wherein females appear to have greater antibody secretion, higher basal immunoglobulin levels, and higher B cell counts (22).

### 4.2 The X chromosome

In addition to the aforementioned factors, male and female infectious responses are directly linked to genetic, biological, and behavioral differences, which include previous exposures to pathogens and sex hormones (20, 34). However, the stronger response to infections among females is also described in the earlier stages of life, before sex hormones exert their ultimate effects, indicating that sex chromosomes and other baseline differences could offer additional explanations for immunological divergence (35). Autoimmune disease susceptibility is also relatively higher among females even before puberty, although the differences between the sexes are less apparent (24). The X chromosome contains at least 50 genes with well-known immune-related functions, including some important for immune cell identity (FOXP3), cellular activation and intracellular signaling (CD40LG, TLR7, IRAK1, IL13RA1/2, NEMO, TASL, IL-9R), leukocyte trafficking (CD99, CXCR3), immune cell differentiation and proliferation (IL-2RG, BTK), and cellular metabolism (OGT, CYBB) (20). Although most alleles on one of the X chromosomes are randomly silenced during female embryogenesis, a subset of genes escape this inactivation. X-inactivation escape results in a gene dosage difference between women and men, which is likely a major factor underlying immune function differences between the sexes. Furthermore, the polymorphism of X-linked genes, cellular mosaicism for X-linked parental alleles, and X-linked miRNA upregulation of several proteins have been suggested to account for immunological sex differences and potentially to create advantages for women in terms of improved host responses to infectious challenges (36), possibly as a result of the modulation of cellular machinery during innate immune responses (37). A recent study proposed a novel mechanism for the increased risk of autoimmunity in diseases with a female bias. The authors suggested that immune responses in patients with diseases such as SLE resulted in targeting of components contributing to the X-inactivation process (38). The number of X chromosomes also impacts autoimmune susceptibility, as clearly demonstrated by the higher frequency of female-discriminant autoimmune diseases (systemic lupus erythematosus and Sjögren’s syndrome) among individuals with sex chromosome aberrations such as Klinefelter Syndrome (47XXY) and trisomy X (47XXX) (20). However, women with Turner syndrome (45X) also have an elevated risk of developing female-biased autoimmune thyroid disease, which may seem paradoxical given their single X chromosome karyotype (39).

### 4.3 The Y chromosome

Despite well-established evidence highlighting the strong role of the X chromosome in regulating immune-related genes, the Y chromosome does have some regulatory influence in shaping immune characteristics in males, as well as being a source of male-specific genetic governance. Evidence for this impact has been gained from different approaches, including murine models of chromosome Y deficiency, detection of chromosome-specific immunoregulatory effects, and examination of manifestations in human males with loss-of-Y. For instance, a mutant mice strain was found to demonstrate Y-linked immunodeficiency involving B and NK cell depletion without an impact on T cells (40). It may be tempting to associate such attributes to hormonal alterations that could confound the analyses; however, it has been demonstrated in coxsackievirus-infected mice that the reduced survival attributed to Y chromosome polymorphism was unassociated with testosterone levels (41), indicating a non-hormonal regulatory role for the Y chromosome on immune response (41). In men with loss-of-Y, fibrotic and inflammatory changes involving macrophages have been understood to underlie cardiac injury (42). Furthermore, polymorphisms in the Y chromosome have been associated with transcriptional changes in macrophages and CD4 T cells, which may be associated with allergic encephalitis and multiple sclerosis, respectively (43).

### 4.4 Infectious susceptibility

The disparity in infection response has most recently been shown by the COVID-19 pandemic (44), with higher estrogen levels yielding better outcomes among patients (45). It is however crucial to note that while male bias regarding susceptibility to infectious disease is almost universally true for the great majority of infectious agents (46, 47), females may suffer from relatively greater disease burden and severity when exposed to certain infections, including influenza, *Legionella pneumophila*, and *Toxoplasma gondii* (48, 49), which might be associated with estrogen levels based on studies showing lower severity before puberty (50) and also weakened immune response in pregnancy (51). However, lower susceptibility is likely to translate into lower overall disease burden in the female population. Testosterone, on the other hand, causes a broad suppression of defensive responses and may ease the spread and impact of different types of infections, including parasitic diseases (46, 52) and uropathogenic *Escherichia coli* (53), among others. Of note, despite the much higher frequency of urinary tract infection in females and the higher proportion of *Escherichia coli* isolation in female patients compared to males, estrogen has been suggested to alter virulence and neutrophil responses which come together to improve the clearance of this pathogen in females (54). Furthermore, androgen exposure in mice has been shown to restrict the phagocytic prowess of neutrophils attracted to the site of infection, plausibly linked to their stunted maturation (55) and exemplifying a direct impact on innate immunity. This limited functionality of neutrophils in the infectious microenvironment has been replicated by other research, this time showing that a higher number of neutrophils are drawn to the site of infection in male mice (further aggravated by testosterone administration), presumably to counteract their limited capacity to clear the infection due to immaturity and limited functionality (56).

### 4.5 Vaccine efficacy and adverse effects

Immunological sex variations are also evident in terms of vaccine efficacy and side effects. Females have better seroconversion but also a higher likelihood of adverse events following immunization, especially local adverse events and allergic reactions (47, 57, 58). The initial response to vaccination is the innate immune system recognizing the non-self, leading to localized inflammation at the injection site and possibly activating allergic response pathways, including anaphylaxis. This is followed by adaptive immune activation which may be boosted by estrogen presence, conferring an advantage for better vaccine response. As such, the female immune system can create a stronger and possibly longer-lasting antibody repertoire after receipt of various vaccines, including smallpox, yellow fever, influenza, and hepatitis A and B (6).

## 5 Immune differences throughout life and sex hormones

### 5.1 Early impacts

**Figure 2:**
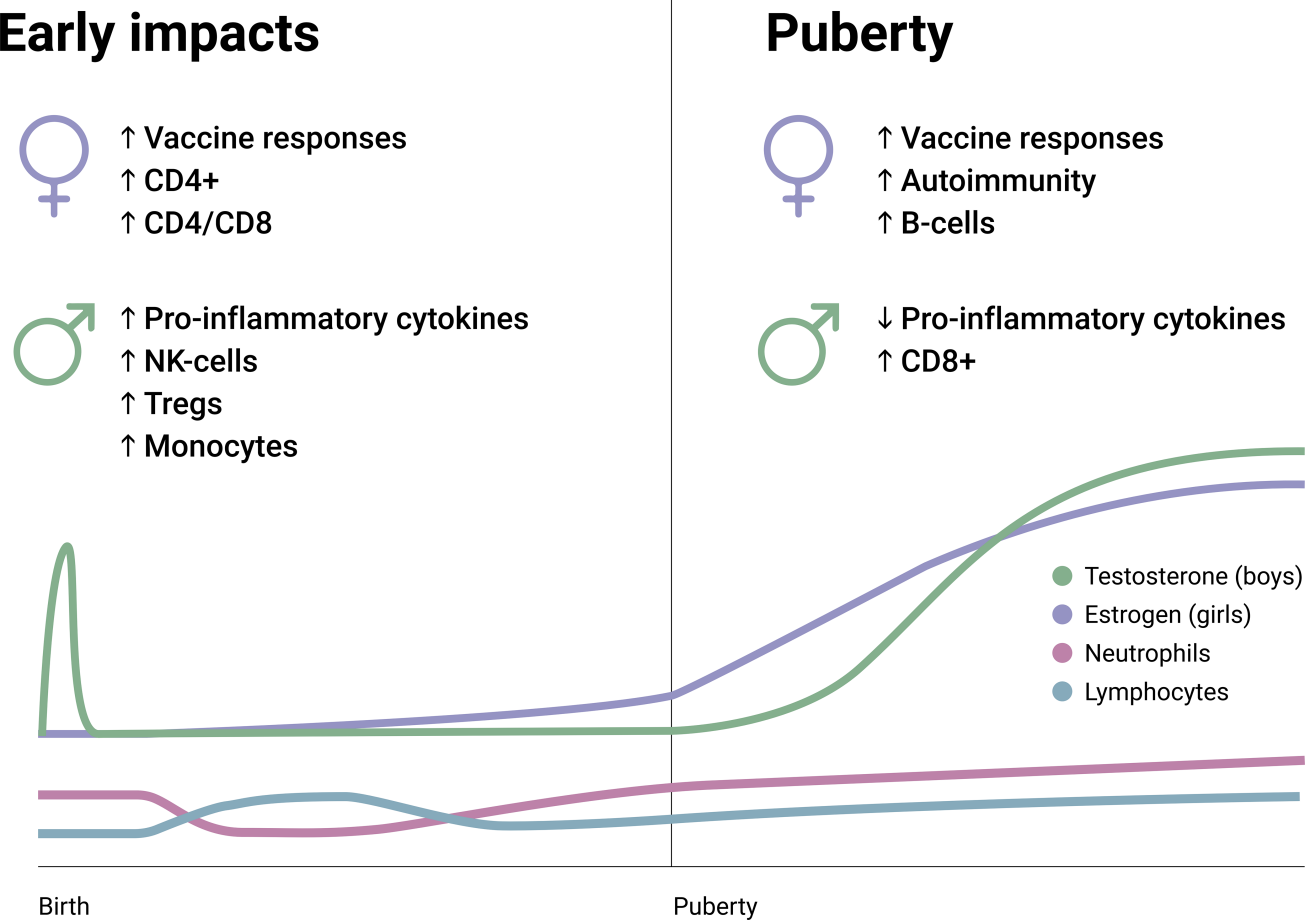
Early and pubertal differences regarding immune constituents and characteristics between boys and girls. Also, pubertal hormone changes (testosterone and estrogen) and the changes in neutrophil and lymphocyte frequencies are summarized on an arbitrary longitudinal scale. CD4+, Cluster of differentiation 4; CD8+, Cluster of differentiation 8; NK, Natural killer; T regs, T regulatory cells.

Apart from the ‘mini puberty’ during infancy, sex hormone production lays dormant throughout childhood (59), with the primary exceptions being gradual increases in anti-Müllerian hormone (in females) and inhibin B (in males). In infants, a higher overall leukocyte count is observed regardless of sex, which necessitates age-specific reference intervals for clinical purposes (60). In terms of cell types and their abundance in the circulation, there are two ‘flips’ between neutrophils and lymphocytes throughout life, both within the early childhood. Data from postpartum studies and longitudinal data collection show that neutrophil count is generally higher compared to lymphocyte count in neonates until around 1-2 months, but lymphocytes prevail as the most common cell type from thereon until 3-5 years of age –when neutrophils regain the lead (61–65). This second ‘flip’ in the neutrophil-to-lymphocyte ratio is postulated to reflect immune maturation and the lifelong balance between the activities of the innate and adaptive immune systems. Moreover, the cytokine profiles also indicate an inversion around 1-3 years of age from a largely anti-inflammatory potential to a vigilant preparedness to produce and secrete pro-inflammatory cytokines (66, 67). These age-related variations during early childhood appear to lose clinical relevance quite swiftly, giving way to the dormancy period of sex hormones in which the great majority of immunological parameters are similar in boys and girls (**Figure 2**).

The early development of the immune system is greatly influenced by environmental exposure as the immune system begins recognizing threats and mounting responses of its own after birth, giving rise to multidimensional variations in relation with the bi-directional relationship between microbiome and immune development (68). The fetal immune system prefers the generation of regulatory T cells which suppress the differentiation and activities of other T cells (69). This might be an adaptive function that facilitates environmental tolerance and might also be associated with the absence of threats. The neonate has susceptibilities to infections and allergies, which are explained by the naïve adaptive immune system and the favoring of Th2 responses rather than Th1 – in correlation with the levels of corresponding cytokines that reveal an anti-inflammatory profile (70–72). Possible regulatory effects have been shown for microbial exposure (68, 73), while breastfeeding and other environmental exposures appear to salvage Th1 response to some degree (74). Furthermore, neonates have rudimentary cellular responses due to limited cytokine production and immaturity in antigen presentation. Repeat challenges to the early immune system generally result in weaker reactivity, a phenomenon recognized as ‘neonate tolerance’ that can underlie lifelong tolerance to select antigens (75), which might be beneficial in the autoimmunity and microbiome contexts but not in terms of initial infectious defense. Indeed, the young innate immune system appears to fail in mounting a sufficient response to pathogenic bacterial challenge (76, 77), creating a predisposition to sepsis (78). This outcome is partially explained by limited TLR-based activation despite the intactness of the machinery required to mount such response (76, 79). Anti-viral and vaccine responses carry a similar hindrance: type I, II, and III interferons suffer an impediment due to dysfunctional signaling that cannot be attributed to lack of response elements (72, 80, 81). These unique regulations involving different aspects of immunity add a functional dimension to the limitations posed by quantitative deficiencies in the cellular components of the immune system in neonates and infants.

Although natural development gives way to more adult-like immunoreactivity profiles in growing children, T cells still appear to have limited inflammatory activation until adulthood (82). Infants continue to manifest early immune system characteristics which may remain relatively dominant well into school age and even puberty (65), but the immune system shows considerable maturation throughout this period (83), particularly with the acceleration of baseline pro-inflammatory potential and the decay of the anti-inflammatory ‘buffer’ of cytokines (67, 84). This maturation is exemplified by the generation of lifelong immunity following vaccination or exposure to childhood infections among school-age children; whereas, antibody levels created by earlier vaccinations are known to diminish quickly (85). Nonetheless, older infants and school age children appear to possess adult-like expression of response elements, including HLA and TLR4 (67), with relatively higher levels of TLR4 –but not TLR2– in boys (86).

### 5.2 Puberty

At the onset of puberty, a complex set of biochemical changes occur in fairly quick succession. While the exact triggering mechanisms are incompletely understood, the immediate origin of pubertal hormonal changes is the pulsatile release of gonadotropin releasing hormone from the hypothalamus, which induces downstream effects on the pituitary, and in turn, the gonads (**Figure 1**) (87). The systemic response differs based on sex, and males emerge with a testosterone-dominated sex hormone profile, while estradiol and progesterone are induced at far greater levels among females (88, 89). This system is called the hypothalamic-pituitary-gonadal axis, which primarily contributes to reproductive development; whereas, in parallel, the hypothalamic-pituitary-adrenal axis also gains increased functionality during puberty and is closely associated with the development and maturation of immune characteristics. There are very few studies that have explored the immunological impact of sex hormones in this specific period, so there is a paucity of specific data in this regard. Males and females also differ in terms of the age of puberty onset and its length, with female puberty beginning around 10-11 years of age and reaching maturity by 15-16 years, while male puberty begins later (11-12 years) and maturity may be delayed (17-18 years) –which increases the complexity of studying this period in relative terms (90). However, available evidence points to a gradual progression towards the immunological characteristics of adulthood in the pubertal period, with few exceptions (**Figure 2**).

Puberty can trigger the onset of autoimmunity by inducing the expression of androgen-regulated autoantigens. A study of patients with autoimmune polyendocrine syndrome type I, caused by biallelic loss-of-function mutations in the *AIRE* gene, identified the prostate-specific enzyme transglutaminase 4 as a major autoantibody target (91). Autoantibodies to this androgen-regulated protein were unique to post-pubertal male patients and longitudinal follow-up revealed that they were triggered by the pubertal onset of transglutaminase 4 expression. Interestingly, the finding of transglutaminase 4 autoantibodies in a single female patient was attributed to receipt of androgenic treatment, which might have triggered transglutaminase 4 (91).

A study involving pubertal and post-pubertal cisgender and transgender individuals as well as post-pubertal patients with juvenile systemic lupus erythematosus reported significantly higher regulatory T cell counts in post-pubertal cisgender males compared to post-pubertal cisgender females (92). In recipients of GAHT, regulatory T cells were found to experience significant shifts that resembled the transcriptomic differences between cisgender males and females, indicating that sex hormones indeed exert ambivalent effects on immune characteristics during puberty. Crucially, the sex-related differences in regulatory cell counts disappeared, but transcriptomic differences were replicated in the subset of patients with juvenile systemic lupus erythematosus. The authors attributed this to a possible dysregulation of sex hormone signaling in the presence of autoimmune pathologies (92).

#### 5.2.1 Female puberty

During female puberty, the menstruation-related fluctuations in estradiol, follicle-stimulating hormone and luteinizing hormone begin, triggering secondary sex characteristics. In a study evaluating peripheral blood mononuclear cells (PBMCs) before and after puberty, estrogen was suggested to be associated with differential methylation of numerous immune-regulating DNA regions in pre- and post-pubertal PBMCs (93). Such variations are also observed during the shorter periods in menstruation. For instance, regulatory T cells with FOXP3, CD4 and CD25 positivity are increased during the estrogen-dominated period of menstruation but decreased following ovulation, indicating a direct impact of estrogens in circulatory cell composition and immunity (94). Taking into account the gradual settling of the menstrual cycle into its adult characteristics during the early stages of puberty, it is difficult to attribute the differences from males to a single factor or hormone. However, population-wide analyses of circulatory cells may shed some light into the development. For instance, adolescent females are described to have relatively higher levels of eosinophils and lower levels of monocytes compared to males of similar age (95). A relatively recent population-based study on this topic intriguingly showed higher eosinophils in males and younger individuals, and male sex retained its significance even after multivariable adjustment for many potentially-confounding factors—including age (96). The low monocyte levels in female adolescents perhaps foreshadow their relatively limited activity/cytotoxicity in adult females compared to males (64, 95, 97). Females have a considerably higher risk for the great majority of autoimmune diseases, which often show a striking increase in incidence following menarche (98–101), indicating the impact of hormones and particularly the rise of estrogen. Furthermore, premature puberty has also been associated with higher likelihoods of autoimmune thyroiditis (102) and multiple sclerosis (100).

#### 5.2.2 Male puberty

Testosterone levels increase at three distinct time points in males: during the prenatal, neonatal, and pubertal stages. The former two peaks are accepted to facilitate the development of the male reproductive tract. During male puberty, androgens promote secondary sexual characteristics and, critically, trigger spermatogenesis (103). Observational studies focusing on pubertal changes in androgens show that boys experience a steep rise in testosterone levels at and throughout puberty, usually maintaining the upward momentum until 16–18 years of age followed by a plateau that often extends to middle age (89, 104). Despite limited data regarding direct relationships with pubertal hormones, the monocyte abundance in males appears to be consistent throughout life (64), suggesting underlying mechanisms other than sex hormones. Additionally, males appear to experience a marginal delay in reaching leukocyte compositions characteristic of adults (particularly with respect to regaining neutrophil dominance) (61–64). This may be associated with the prolonged puberty period and continuous rise in testosterone levels throughout puberty. In a longitudinal study exploring genetic features during puberty, analyses showed that males had a considerably larger number of differentially-expressed genes relative to females (105). Taken together with the divergence of DNA methylation features during puberty (93) and the varying impacts of estrogen and testosterone in this regard (106), the onset of puberty appears to have the potential to at least partially shape the underlying properties associated with immune response. In this context, studies exploring DNA methylation in males and females have revealed numerous differences that may have physiological and pathological implications, including generally higher levels of autosomal methylation in females (107), differentially-methylated regions that associate with sex hormones (108), and variabilities in immune response to cancer (109).

### 5.3 Adulthood

**Figure 3:**
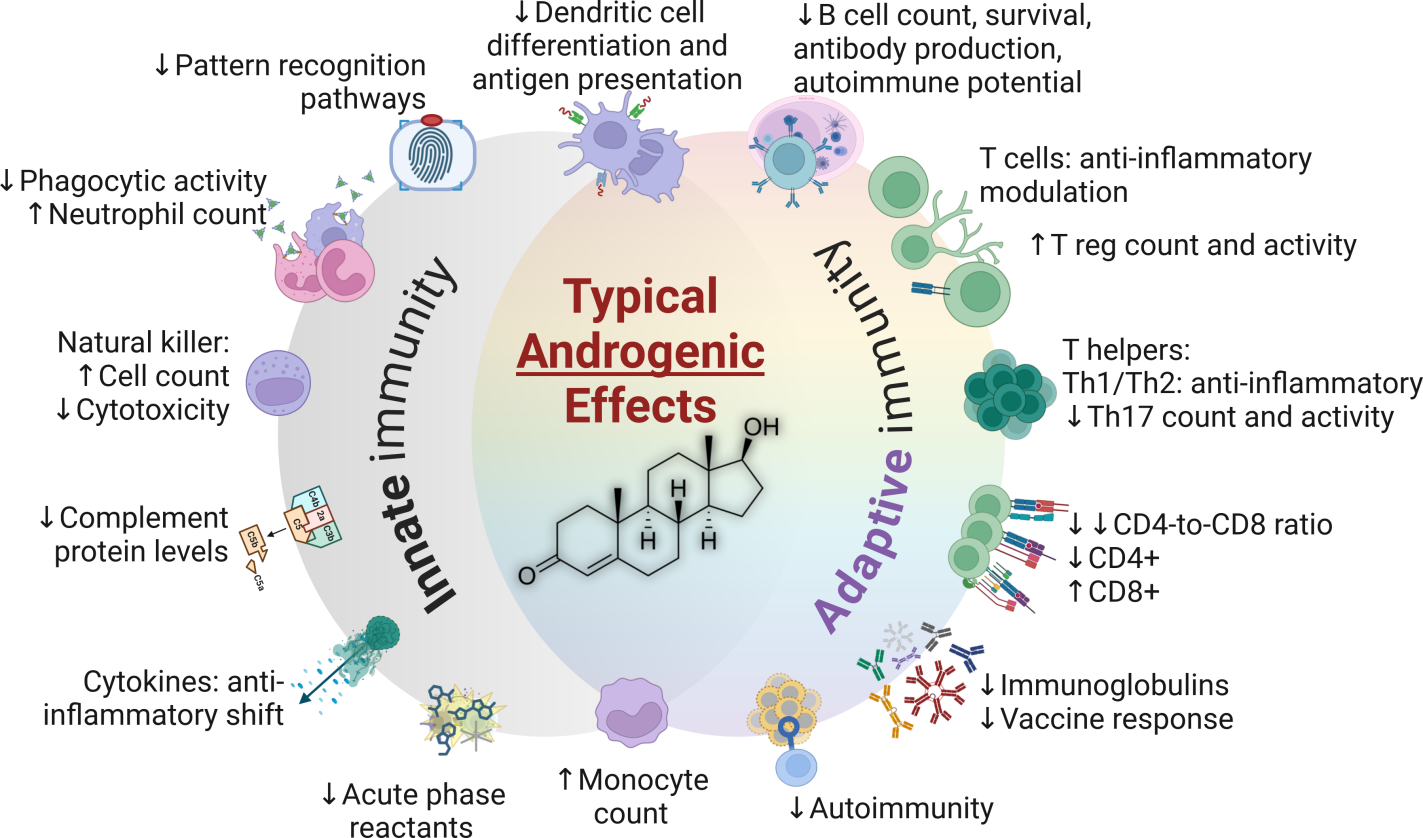
The typical impacts of androgens in the immune system (testosterone structure depicted in center). CD4+, Cluster of differentiation 4; CD8+, Cluster of differentiation 8; Th, T helper cells; T reg, T regulatory cells.

**Figure 4:**
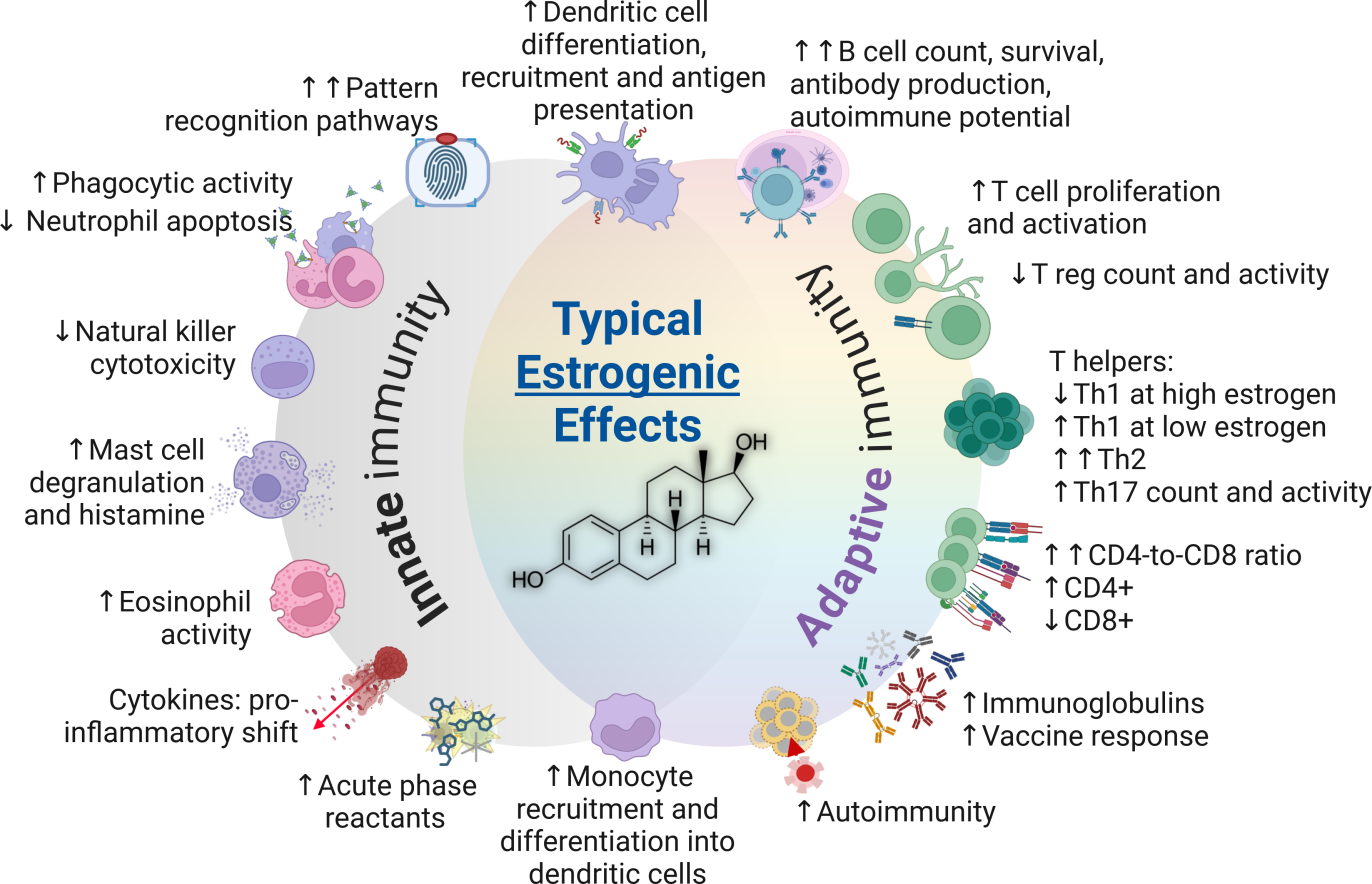
The typical impacts of estrogens on the immune system (estradiol structure depicted in center). CD4+, Cluster of differentiation 4; CD8+, Cluster of differentiation 8; Th, T helper cells; T reg, T regulatory cells.

Sex hormones exert their most discernable impacts on immune properties and functions during the adulthood. A comprehensive summary of innate and adaptive immune characteristics in adults is presented in **Table 1**. Furthermore, the typical impacts of androgens and estrogens are summarized in **Figures 3**, **4**, respectively.

**Table 1:** Immune dynamics throughout adult life.

	Male	Female	Important notes for autoimmune or inflammatory diseases	Pregnancy (mostly relative to adult female)	Menstruation (mostly relative to adult female)	Andropause & testosterone loss (mostly relative to adult male)	Menopause & estrogen loss (mostly relative to adult female)
Innate							
Pattern recognition	↑ TLR4 expression in neutrophils (110). ↑ TLR2 response upon stimulation (111).	↑ TLR3, TLR7, TLR9 overall expression and response (111–114). ↑ TLR2 expression in monocytes (115). ↑ Response to TLR7/8 stimulation (116).		↓ TLR2 expression in the cervical epithelium of subjects with miscarriage (117).		↑ TLR4 after orchiectomy in mice (118). ↑ TLR2, ↓ TLR7, ↓ TLR8 in the ocular surface of older (>61 years) healthy subjects (119).	↑ TLR2, ↓ TLR7, ↓ TLR8 on the ocular surface of older (>61 years) healthy subjects (119).
Phagocytes and phagocytic activity	↑ TNF production in neutrophils (110). ↓ Circulating NOx levels (120).	↑ Phagocytic activity of neutrophils and macrophages (121). ↑ Antigen presentation (122).		↑ Immature cells (123). ↓ Inhibitory effect on neutrophils (124).	↓ Neutrophils in follicular phase vs luteal phase (125).	↓ Neutrophils in orchiectomy vs testicular cancer (126).	↓ Neutrophil absolute count (127). ↓ Innate cytotoxicity (128–130).
Natural killer cells	↑ Absolute count (33).	↑ Activation (cytotoxicity and degranulation upon IFNα stimulation) (131).		↓(slight) Circulation; ↑ decidua (132). ↑ Relative in repeated miscarriage (133). ↓ Cytotoxicity after challenge (exceptions exist) (134–136).	↑↑ Count in mid and late luteal vs follicular (137). ↑ (Slight) cytotoxicity in mid luteal vs follicular (137).	↑ Relative with androgen deprivation (138). ↑ Following orchiectomy (139). ↑↑ Absolute count (140).	↑ Absolute count (140, 141). ↑ Percentage in premature menopause vs similar-age non-menopause (142) * Stronger cytotoxic response in elderly females vs elderly males (both >70 years) (143).
Mast cells/Eosinophils/Basophils	↑(slight) Eosinophil count (144).	↓(slight) Eosinophil count (144). ↑ Mast cell activity, degranulation (145).	↑ Eosinophil count in asthmatic males (↑↑ boys vs girls) (childhood onset flips the relationship in adulthood) (144, 146).	↔ Eosinophils and basophils throughout pregnancy (147).			
Complement system	↑ C3 and properdin levels (148). ↓ Factor D (C3bB cleavage) (148).	↓ C3 and properdin levels (148). ↑ Factor D (C3bB cleavage) (148).	↑ C5 levels in synovial fluid in osteoarthritis (149).	↑ C3 and C4 levels, gradual increase throughout pregnancy (150).	↑ C3 levels in luteal phase vs follicular (endometrial tissue) (151).		↑ C3 (152). ↔ C4 (152). ↓ C3 and C4 in non-ERT compared to ERT recipients (153).
Cytokines/Chemokines	↑ IL10 upon TLR8/TLR9 stimulation (154) ↑ IL1β and IL6 production of macrophages upon TLR4 stimulation (155). ↑ TNF production in PBMC upon TLR4 stimulation (156).	↑ IL6 following bacterial challenge (156). ↓ TNF following bacterial challenge (157). ↑ CCL20 (limited male data) (158). ↑↑ IL10 following bacterial challenge (157).	↑ IL17 and IL23 in females with RA (but also higher anti-inflammatory TGF-β) (159). ↑ IL17 in male patients with AS but not females (160). ↓ IL4 in RA; ↓↓ in females with RA (161).	↑ IL4 (162). ↓ T1 and T3 interferons (163, 164).	↑ IL6 in early follicular vs early luteal (165). ↑ TNF early follicular (166). ↓↓ IL1β, TNF, IFNG, NFKB1, TGFβ gene expression in mid-follicular phase vs other phases (167). ↑ IL10 in follicular vs early luteal following bacterial challenge (157).	↓ IFNG expression (138). ↑ IL1β, IL6, IL10 and TNF (168, 169). ↑ TNF after orchiectomy in mice (170). ↔ ICAM-1 and VCAM-1 (171). * Negative correlation between testosterone and soluble IL6 receptor (169).	↑ Pro-inflammatory (172). ↑↑ CCL20 (158). ↑(slight) TNF (173).
Acute-phase proteins	↓ or ↔ overall inflammatory activity (testosterone-mediated) (174). ↑ Albumin and transthyretin (negative acute-phase proteins) (175).	↑ CRP (176). ↓ Albumin and transthyretin (negative acute-phase proteins) (175).		↑ Erythrocyte sedimentation rate (177). ↓(slight) Ferritin (147). ↑↑ Early postpartum (178, 179).	↑ CRP early follicular (166).	↑ CRP in hypogonadal males (180). ↑ CRP in older men (65 to 85+ age) (169). ↔ CRP in partial androgen deficiency (171, 181).	
Adaptive							
B cells	↓↓ Circulating, transitional, and mature cells (182). ↓ Survival (182). ↓ Germinal center migration (autoimmune potential) (183).	↑ Overall function and count (184, 185). ↓ Lymphopoiesis (186). ↑↑ Maturation (autoimmune potential) (183).		↔ Overall count; ↓ B regs (187). ↓ Most subsets (except naïve) in the third trimester (188).	↔ Throughout menstruation (137).	↔ or ↑(slight) after orchiectomy (139).	↔ or ↓(slight) (141, 142, 189). * Higher circulatory cells in elderly females vs elderly males (>70 years) (143).
T cells general	↓ (Particularly downward trend with age) (190).	↑(Slight) cytotoxic cells (190).		↓ Overall count (132). ↑ Immature cells (123).		↑ Naïve T cells following androgen deprivation (191). * Greater declining trend with age (190).	↓ Absolute and relative count (141, 142). ↑ Th1/Th2 ratio (141). ↑ Th17/Treg ratio (141).
T regulatory	↑ Cell count and percentage (92, 192). ↑↑ Cell count in visceral adipose tissue (193). ↑ FOXP3 expression (194). ↔ Estrogen-mediated CD4, CD25, FOXP3 expression in healthy males (194).	↓ Cell count and percentage (92, 192, 194). ↓↓ Cell count in visceral adipose tissue (193). ↓ Immunoreactivity (195). ↑ Estrogen-mediated CD4, CD25, FOXP3 expression (194). ↑ Testosterone-mediated FOXP3 expression (194)	↓↓ Cell count (SLE) (194). ↑ Treg FOXP3 expression in females with RA (159). * Differentially regulated in SLE (194).	↑↑ Decidua (196). ↑ T reg count and percentage during pregnancy (132, 197–199). ↓ Cell count in unexplained infertility (200) and miscarriage (198). ↓ FOXP3 expression (187).	↑ Follicular phase; ↓ after ovulation (94). * Females in the late follicular phase have similar T reg percentage to males (94).	↓ Following androgen deprivation (138).	
T memory	↑ Count and relative, including CD4 or CD8 positive memory stem, central memory, and effector memory cells) (201).	↓ Count and relative, including CD4 or CD8 positive memory stem, central memory, and effector memory cells (201).		↓ Cell count (132).		↓ Following orchiectomy due to testicular cancer (202).	↑(slight) upward trend in central and effector memory cells with age (particularly >50-60 years) (201). * Lower circulatory CD4+ memory cells in elderly females vs elderly males (>70 years) (143).
T helper 1	↓ But ultimately favoring Th1 relative to Th2 (23).	↑ With low estrogen; ↓ with high estrogen (203).	Similar in males and females with RA (159). Similar in males and females with AS (160).	↓ Overall response (132).	↑ Response in late luteal phase (7).	↑ Response with androgen deprivation (191).	
T helper 2	↓ Overall response (204).	↑ Overall response (23). ↑↑ With high estrogen (205).		↓ Overall response (132).	↑ Response in follicular phase (6)		↓ IL10-producing Th2 (141).
T helper 17	↓Cell count and activity (25). ↑ Immunoreactivity (autoimmune potential) (195).	↑ (via ERα) (124). ↓↓ (via ERβ) cell count and activity (25, 206). ↓ (via progesterone) differentiation (207).	↑ Cell count and activity (general) ↑ In male patients with RA (159). ↑ In male patients with AS but not females (160).	↓↓ Cell count and activity via estrogen and progesterone (197). ↑ Cell count in unexplained infertility (200).			↑ Absolute and relative count (141).
CD4+	↓↓ (33).	↑↑ (33).		↑ CD4+ T reg percentage in the first and second trimester (198, 199).	↑ CD4+ T reg count and FOXP3 expression in late follicular phase; ↓ in early follicular and late luteal (94).	↓ Lower count in androgen deprivation (138).	↓ Circulation count (141, 142). ↓ Function in the reproductive tract (208). ↓ Premature menopause vs similar-age non-menopause (142)
CD8+	↑ Absolute count (33).	↓ Absolute count but ↑ cytotoxicity (33, 209).		↑ CD8+ T reg percentage continuous rise throughout pregnancy (198). ↑ fetal-specific CD8+ T cells (210).		↓ Following orchiectomy due to testicular cancer (202).	↓ Absolute count (141). ↓↓ Relative (percentage) (141). ↑ Premature menopause vs similar-age non-menopause (142).
CD4/CD8 ratio	↓↓ (33).	↑↑ (33).				* Inversion of ratio (higher) (211). ↔ androgen deprivation (138).	* Inversion of ratio (lower) (172, 211).
Immunoglobulins	↓ Especially IgG and IgM (182).	↑ Overall levels in circulation (186) ↑ IgM production (212).	↑↑ IgE levels in asthmatic males vs asthmatic females (childhood and adulthood) (146).	↑ Antibody production (213).			
Vaccine response (efficacy)	↓ (214).	↑ (214, 215).		↔ (216, 217)		↓ (218, 219).	↓ or ↔ depending on vaccine type and dose (215, 220).
Vaccine adverse effects	↓↓ Frequency and severity (220, 221).	↑↑ Frequency and severity (220, 221).		↔ (216, 217)			↔ (220)
Autoimmunity and autoantibody production	↓↓ Especially pathological autoantibodies (182).	↑ Autoantibody levels and frequency (22)	↑ Higher frequency of atopy in asthmatic males vs asthmatic females (childhood and adulthood) (146).				↔ (189). ↓ Atopy (146).

↓: Decreased

↑: Increased

↔: No significant change or stable

*: Relevant note

Abbreviations in alphabetical order: AS, Ankylosing Spondylitis; B regs, B regulatory cells; CCL, Chemokine (C-C motif) ligand; CD4+, Cluster of differentiation 4; CD8+, Cluster of differentiation 8; CRP, C-reactive protein; ERT, Estrogen replacement therapy; ERα/ERβ, Estrogen receptor alpha/beta; FOXP3, Forkhead box P3; ICAM-1, Intercellular adhesion molecule 1; IFN, Interferon; IgE, Immunoglobulin E; IgG, Immunoglobulin G; IgM, Immunoglobulin M; IL, Interleukin; NFκB, Nuclear factor kappa-light-chain-enhancer of activated B cells; NK cells, Natural killer cells; NLR, NOD-like receptor; NOx, Nitric oxide metabolites; PBMC, Peripheral blood mononuclear cells; RA, Rheumatoid arthritis; SLE, Systemic lupus erythematosus; TGF-β, Transforming growth factor-beta; Th cells, T helper cells; TLR, Toll-like receptor; TNF, Tumor necrosis factor; Treg cells, T regulatory cells; VCAM-1, Vascular cell adhesion molecule 1.

#### 5.3.1 Female immunity and estrogens

Estrogens enhance immune response, with the sole exception suggested to be a mild-to-moderate decrease in cell-mediated immune responses (18). However, they also contribute to a higher vulnerability to autoimmune diseases (98–100). Estrogens promote Th2 responses and stimulate antibody production, with evidence showing an increase in regulatory T cells during the follicular phase of the menstrual cycle when estrogen levels peak (94). In terms of Th1, estrogens are accepted to yield overall promoting effects at low concentrations and suppressive effects at high concentrations (7). Estrogen improves neutrophil responses and decreases their apoptotic potential (127) and activates B lymphocytes (222). Although cellular senescence is associated with weaker immune response (223), delayed apoptosis could extend the life of mature immune cells, which may improve immunoreactivity during infection but could also increase the likelihood of autoreactivity (224). Therefore, the impact of estrogen appears to be advantageous in the context of infectious response but detrimental for classical autoimmune diseases in adults (225). Estrogen receptors are present in the immune system with varying expression in different cell types, influencing both innate and adaptive immunity as demonstrated by the transcriptional and protein-level data obtained from dendritic cells, T and B cells, and monocytes (226).

##### 5.3.1.1 Menstruation

It is also crucial to consider the fluctuations in estrogen levels that occur throughout life. Before menopause, females typically have about five times the circulating estrogen levels of males (227), which is accepted to be the underlying factor causing Th2 activation and Th1 suppression (6). However, levels can vary significantly with the menstrual cycle and some females experience short periods when their estrogen levels decline to male levels. These periods are typically during the luteal phase of the menstrual cycle and lead to activation of Th1 responses (7), mild inflammation, and marginal elevation of specific inflammatory markers, such as C-reactive protein (228). Furthermore, a meta-analysis of 110 studies examining menstrual correlations with constituents of the immune system revealed that the follicular phase (higher estrogen) was associated with lower counts of circulatory immune cells, including leukocytes, neutrophils, and particularly, monocytes (125) – notably with very high heterogeneity (*I^2*) among results. Other studies have shown stable B cell populations during menstruation (137), higher regulatory T cells in the follicular phase relative to luteal (94), and higher NK cell count in the luteal phase relative to follicular (125, 137).

##### 5.3.1.2 Pregnancy

Pregnancy also influences the immune system by triggering a shift toward Th2 response and increased antibody production, while general immunoreactivity declines with higher expression of anti-inflammatory cytokines, ultimately resulting in lesser severity of many but not all forms of autoimmune conditions (213). This notion is supported by the fact that increasing estrogen and progesterone levels during pregnancy would be expected to suppress Th17 cells (25), which has been described previously (197); however, contrasting results showing increases in Th17-associated cells in pregnant women exist (132). The latter study also showed lower circulatory lymphocyte levels, lower Th1/Th2 memory cells, and progressive declines in regulatory T cells during pregnancy (both in absolute and relative terms) when compared to non-pregnant women (132). Although the great majority of immune cells rebounded to non-pregnant levels very swiftly following delivery, it was notable that the memory cells did not demonstrate this swift recovery, which is an interesting aspect that warrants further study (132). NK cells are among the immune cells that strongly deviate during pregnancy (229), both in the circulation and pregnancy decidua. While the abundance of NK cells in the decidua is recognized to facilitate placental development, the cytotoxic subset of NK cells may decline in maternal circulation (132) which facilitates implantation (230); however, available NK cells appear to mount stronger responses to viral challenge (specifically influenza) (229) in parallel with the pro-inflammatory profile of monocytes and dendritic cells in pregnant women with influenza (231). Nonetheless, suppression of the cytotoxic potential of NK cells have been shown in other types of stimulation, and it must be mentioned that NK-deficient mice appear to experience better outcomes when infected with influenza (134, 135). Taken together, pregnancy is a period when the female immune system mounts relatively weaker immune responses and less autoimmune potential that correlate with rising estrogen levels and NK cell levels. This change also appears to be responsible for elevated viral susceptibility (232) that is also partially explained by diminished type I and type III interferon responses reported by experimental studies (163, 164).

##### 5.3.1.3 Menopause and estrogen loss

Menopause is characterized by the natural loss of estrogen synthesis (233). Coinciding with the fall in endogenous estrogen levels, the advantage conferred to females in terms of infectious diseases is largely lost (37), while the severity of autoimmune diseases lessens (234, 235). Innate immunity-related changes include declines in anti-inflammatory cytokines, neutrophils and cytotoxic potential, while pro-inflammatory mediators and effectors demonstrate a general increase (127, 172). The silencing of estrogen signaling mirrors an inversion of the CD4-to-CD8 lymphocyte ratio, weaker vaccine response, and considerable variations in T cell populations (141, 211). In systemic lupus erythematosus, the female bias is exceedingly apparent (10–15:1), similar to multiple sclerosis, Sjögren’s and other autoimmune disorders (99, 236). However, estrogens are not the only explanation to this difference, as overt autoimmunity is observed at a higher frequency among pre-pubertal females and the female bias persists after menopause (99, 236–238).

Hysterectomy and oophorectomy are procedures that result in the resection of estrogen-producing tissues. The outcomes of these procedures also appear to reduce the severity of autoimmune diseases while restricting immune response to microorganisms – as demonstrated by experimental and clinical studies (128–130).

#### 5.3.2 Male immunity and androgens

Androgens modulate the innate immune response through a number of mechanisms, including cell proliferation, cytokine secretion, and the expression of pattern recognition receptors such as toll-like receptors (TLRs) (103). Dihydrotestosterone is far more potent that testosterone in terms of androgenic effects, but the most abundant androgen in adult men is testosterone (239). Androgens exert mainly immunosuppressive properties (240) through the induction of anti-inflammatory cytokines and suppression of nitric oxide (NO) production in neutrophils, monocytes, and macrophages, acting as a limiter to their cytotoxic potential (120, 241). Testosterone also causes lower relative levels of CD4+ T cells compared to CD8+ T cells, in direct contrast to the overall impact of estrogens (37). The prohibitive impact on polymorphonuclear cells and the innate immune system, as evidenced by anti-inflammatory modulation of monocytes and macrophages (240), is one of the most prominent factors explaining the relatively weak response to infection among males (35).

##### 5.3.2.1 Dehydroepiandrosterone – a unique androgen

Dehydroepiandrosterone (DHEA) is a unique intermediate steroid (prohormone) in that it is recognized by both androgen and estrogen receptors, appears to have divergent impacts on immune functions, and that it can be enzymatically transformed to these steroids through different pathways, depending on tissue exposure. DHEA has weak binding properties to androgen and estrogen receptors, which indicates that its receptor-mediated effects might be overridden or diminished by the presence of androgens or estrogens with higher receptor affinities, and also, that it could exert its effects through other signaling pathways (242, 243). It has been shown to regulate some immune responses, by suppressing the synthesis of proinflammatory cytokines such as IL-2 and IL-6, and stimulating anti-inflammatory cytokines such as IL-4 and IL-10 (244). These properties appear to yield physiologically-relevant impacts as evidenced by milder disease manifestations when administered to females with systemic lupus erythematosus (245–247). Based on studies in humans, DHEA is understood to increase monocyte and NK cell counts (248), restrict IL-5, IL-10 and IFN-γ secretion in patients with asthma (249), negatively correlate with the parasitic burden of malaria (DHEA-S) (250), and demonstrate diminished levels in patients with tuberculosis – similar to decreased testosterone levels (239, 251). Alluding to the contrasting effects of DHEA on immune function in the context of immunopathology, oral supplementation in patients with Addison’s disease was found to decrease NK cells while restoring regulatory T cells (252). Adding to this complexity, under experimental infectious challenges, DHEA appears to stimulate IFN-γ in parasitic infections, thereby promoting response (253) in a striking similarity to estrogen’s impact on IFN-γ (254), and also, it improves macrophage phagocytosis via NO upregulation in bacterial challenge through favoring of Th1 responses in contrast to Th2 (increased IL-2 and IFN-α; decreased IL-4 and IL-10) (255). In a study examining stress responses among males, post-stress DHEA levels were found to correlate positively with the anti-bacterial activity of saliva (256). Some of these ambivalent effects may be associated with the sensitivity of estrogen receptors to DHEA (257) and its stimulation of the NF-kB pathway (244), as well as its multiple effects on other pathways (243). Therefore, although DHEA indeed has similar immunomodulatory effects with other androgens, such as testosterone and dihydrotestosterone, its contrasting effects must be appreciated when interpreting the influences of this unique prohormone on immune functions, which remain limitedly understood (239).

##### 5.3.2.2 Andropause and testosterone loss

While menopause is a clear threshold with which it is possible to explore the impact of a sudden decline in estrogen levels on female immunity, males do not have such a period with an abrupt loss of testosterone levels, but they experience a very gradual and plateau-like testosterone decrease after middle age (258), which can result in andropause in elderly men. Supportive evidence regarding the impact of testosterone on autoimmunity development (259) can be drawn from studies showing elevated autoimmunity risks among patients with hypofunctional testes (260). Data obtained from orchiectomy studies provide some more context to the matter of immune functions orchestrated by androgens. Loss of testosterone production due to orchiectomy has been associated with an increase in TNFα production in an experimental study (170), while another murine study revealed that orchiectomy yields macrophages with increased expression of TLR4 and that androgen-naïve macrophages exhibit decreased TLR4 levels in response to testosterone stimulation (118). Furthermore, in a clinical follow-up of patients who had undergone orchiectomy, a significant increase in NK cell count was identified at 3 months after surgery. Notably, the same study revealed an increase in B lymphocytes, but statistical analysis was marginally non-significant (139). Other impacts of testosterone loss include lowered IFNG expression (138), increased naïve T cell counts (191), fewer memory and regulatory T cells (138, 202), a greater propensity towards Th1 responses (191), and inversion of the CD4-to-CD8 ratio (211).

##### 5.3.2.3 Androgens in females

The immunosuppressive impact of testosterone is not limited to males. Females with elevated testosterone levels due to polycystic ovary syndrome have been shown to suffer from more severe COVID-19 relative to those without polycystic ovary syndrome (261) and this relationship appears to be mediated by inflammatory modulation and the facilitation of viral entry to cells (262, 263). One study specifically examining women with and without hyperandrogenism described considerable differences in the frequencies of mild-to-moderate COVID-19 symptoms. Hyperandrogenic women manifested with significantly higher frequencies of anosmia, ageusia, cough, fatigue, anorexia, and pain (264).

### 5.4 Exogeneous hormones and gender-affirming hormone therapy

In addition to natural fluctuations in endogenous hormones, individuals may also experience changes due to receiving exogenous hormones, such as hormonal contraceptives, hormone replacement therapy, and GAHT (233). These treatments are known to impact the homeostasis of various hormonal and metabolic pathways, including the pituitary-adrenal axis (265–267). The impact on this pathway, more so than the alterations of sex hormones, could explain many changes in immune regulation (268). For instance, testosterone and estrogen + antiandrogen therapies respectively administered to transmen and transwomen exerted effects that resembled the typical differences between cisgender males and females in terms of the pituitary-adrenal axis. Transmen experienced a decrease in cortisol production while transwomen had elevated levels (267). Based on decades of research, oral contraceptives have been established to influence a multitude of processes and systems in the body, including coagulation, hormonal homeostasis, energy metabolism, leukocyte counts, and other immunity-related parameters (269, 270). In fact, pubertal use of combined contraceptives has been associated with a decrease in Th17 lymphocytes, albeit the functionality of these cells and the levels of related cytokines were elevated – possibly balancing the overall deleterious effect (271). That being said, the effects of exogenous estrogens are varied. Estrogens alone appear to facilitate an elevation of regulatory T cells in both absolute and relative measures and promote differentiation of several cell populations, such as dendritic cells (272), with some studies reporting reduced inflammation during hormonal contraceptive use (273) while others have reported an increase in inflammatory markers and disease (274, 275). An in-depth review and contextual examination of the primary effects of exogenous sex hormones and GAHT on different diseases has described available evidence and the significant gaps and conflicts in current knowledge (276).

These conflicts may be a result of numerous treatment- and patient-related characteristics; however, the alteration of the underlying physiological ‘norm’ could be a reliable explanation. For instance, estrogen levels exceeding physiological levels have been associated with a dose-dependent effect on immune response in a meta-analysis involving multiple species. The authors revealed that supraphysiological estrogen levels had a moderate enhancing effect on immune response, while physiological levels did not (18). The same study also showed a weak relationship between higher testosterone and immunosuppression (18). Notably, an early study examining the results of anti-androgen treatment administered to transmen revealed that circulatory NOx levels (nitrite + nitrate, emerging from NO decomposition) were increased after 30 days of treatment, and correlated positively with estradiol while DHEA-S declined (120). As such, the expected results of exogenous testosterone and estrogen administration in GAHT may be feasibly aligned with their established immunoregulatory effects. In agreement with this hypothesis, GAHT was found to cause transcriptomic changes in regulatory T cells that mirrored the differences between cisgender males and females (92).

Although research concerning the impact of GAHT is yet in its infancy, exogeneous hormone treatments have long been used in patients with sex chromosome aberrations. For instance, testosterone replacement therapy in patients with Klinefelter’s Syndrome has been described to lower antibody and cytokine levels and lymphocyte counts (both T and B cells) [5]. The approaches to GAHT differ based on individual requirements and also from center to center, which may include the suppression of endogenous sex hormones as well as estrogen or testosterone administration to transgender women or transgender men, respectively. A recent study examining immune adaptations in 23 transgender men undergoing testosterone-based masculinizing treatment revealed various changes in immune cell populations when comparing data from up to 1 year of follow-up to baseline characteristics. The rise in testosterone and subsequent suppression of estradiol was found to downregulate the type I interferon system and upregulate TNF at multiple levels (277). A similar suppressive effect of GAHT on the type I interferon system was observed in an independent study of transgender men (278). Type I interferon suppression could reasonably explain poor viral outcomes in males, while the presence of lower testosterone and higher estrogen may possibly overactivate type I interferons in women, which may add another dimension to the relationship of these hormones with autoimmune diseases such as systemic lupus erythematosus – as evidenced by the aforementioned positive impact of DHEA on disease manifestations (245, 247, 279).

Another study assessing the impact of testosterone-based GAHT treatment on metabolic and inflammatory markers found that testosterone therapy increases leukocyte-endothelium interactions (280). This is attributed to an increase in polymorphonuclear leukocyte rolling and adhesion, along with a reduction in rolling velocity. The treatment also increased the levels of vascular cell adhesion molecule-1, E-selectin, IL-6, and TNFα (280). However, the expected impact of cross-sex hormones may not be as clear for other immune features. This can be exemplified by a study showing that transgender women using transdermal estradiol experienced increased platelet activation and coagulation marker levels, whereas transgender men using testosterone did not show any contrasting alterations in this respect. The authors also reported that inflammatory markers appeared to be diminished among transgender women, while high-sensitivity C-reactive protein levels increased in transgender men (281). Another study in transgender men and women revealed changes in gut microbiome composition following the initiation of GAHT (282). Given the important role of the microbiome in shaping immune function, these shifts may contribute to explaining immunological sex differences and related disease susceptibilities, as previously shown for autoimmune disease manifestations in mice (283).

A few case reports have documented the onset of autoimmune diseases, primarily systemic lupus erythematosus, but also systemic sclerosis, rheumatoid arthritis, and other rheumatic conditions, in transgender individuals undergoing GAHT (284). Anti-nuclear antibodies, which can precede the development of autoimmune conditions, were examined in a recent study where 36% of transmen and 31% of transwomen tested positive, compared to just 13% in the cisgender male and female population (285). This is a remarkable situation particularly for transgender men who would be expected to lose the impact of estrogen dominance on autoimmune disease susceptibility. Nonetheless, the consistently higher positivity for anti-nuclear antibodies in both transgender groups solidifies our understanding that estrogen and testosterone influence endogenous immune control mechanisms in infectious or autoimmune conditions, which is a conclusion supported by other studies revealing elevated anti-nuclear antibody levels among transgender individuals compared to the general population (286). However, it must be noted that the conflicts in the literature also extend to this relationship, as data from another study that prospectively evaluated the presence of autoantibodies among recipients of GAHT for 3 years revealed that the treatment did not yield an increased risk of developing overt autoimmune diseases (287), which could suggest that elevated immunoreactivity might not translate into an appreciable risk of clinical disease. It is also tempting to postulate that these risks might be ameliorated by the governing genetic characteristics underlying the immune functions of transgender individuals. One particular aspect is that transmen lack the Y chromosome, which, as described previously, has regulatory impact on immune function.

While available studies present conflicting results, what remains undisputed is the significance of this research area, which holds the potential to illuminate the existing knowledge gaps in GAHT and the impact of sex hormones on immune functioning. Many screening recommendations exist for transgender individuals undergoing GAHT –including assessments for cardiovascular risk, osteoporosis, breast cancer, cervical cancer, and prostate cancer (288). However, there is a need for more information to determine whether the immunological effects of GAHT and potential impacts on immune-related disease risks also need consideration. Studies evaluating this topic also offer crucial data regarding sex differences in immune function, which may in turn support development of new treatments for immune-related diseases that are better tailored to each sex.
